# Ancestors’ dietary patterns and environments could drive positive selection in genes involved in micronutrient metabolism—the case of cofactor transporters

**DOI:** 10.1186/s12263-017-0579-x

**Published:** 2017-10-04

**Authors:** Silvia Parolo, Sébastien Lacroix, Jim Kaput, Marie-Pier Scott-Boyer

**Affiliations:** 1 The Microsoft Research, University of Trento Centre for Computational Systems Biology (COSBI), piazza Manifattura 1, 38068 Rovereto, TN Italy; 2 Vydiant, Inc, Gold River, CA USA

**Keywords:** Positive selection, Cofactor transport, Inter-individual variability, Ancestry, Dietary habits, Biological response

## Abstract

**Background:**

During evolution, humans colonized different ecological niches and adopted a variety of subsistence strategies that gave rise to diverse selective pressures acting across the genome. Environmentally induced selection of vitamin, mineral, or other cofactor transporters could influence micronutrient-requiring molecular reactions and contribute to inter-individual variability in response to foods and nutritional interventions.

**Methods:**

A comprehensive list of genes coding for transporters of cofactors or their precursors was built using data mining procedures from the HGDP dataset and then explored to detect evidence of positive genetic selection. This dataset was chosen since it comprises several genetically diverse worldwide populations whom ancestries have evolved in different environments and thus lived following various nutritional habits and lifestyles.

**Results:**

We identified 312 cofactor transporter (CT) genes involved in between-cell or sub-cellular compartment distribution of 28 cofactors derived from dietary intake. Twenty-four SNPs distributed across 14 CT genes separated populations into continental and intra-continental groups such as African hunter-gatherers and farmers, and between Native American sub-populations. Notably, four SNPs were located in *SLC24A3* with one being a known eQTL of the NCKX3 protein.

**Conclusions:**

These findings could support the importance of considering individual’s genetic makeup along with their metabolic profile when tailoring personalized dietary interventions for optimizing health.

**Electronic supplementary material:**

The online version of this article (10.1186/s12263-017-0579-x) contains supplementary material, which is available to authorized users.

## Background

Diet and food availability shaped genetic variation in humans and left distinct adaptation signals among geographically and culturally diverse populations [1–3]. Lactase persistence in adults is the prime example of food-based positive selection. Cattle domestication after the Neolithic transition provided access to dairy products and the advantages of an additional source of calories, calcium, protein, and other nutrients [4]. The ability to utilize this nutrient dense food resulted in a strong positive selective pressure on a variant of the lactase-phlorizin hydrolase gene (*LCT*) responsible for lactose metabolism in the small intestine [5, 6]. Other genetic changes can also be selected by food availability. For example, the number of copies of the salivary amylase gene may reflect adaptation to starch-rich diets and with consequences for modern health as amylase copy number variations may be negatively associated with body mass index [7–9]. Positive adaptation signals have also been described for *FADS2*, which codes for an enzyme involved in long-chain polyunsaturated fatty acid synthesis. A variant of *FADS2* was associated with higher mRNA expression in vegan individuals [10] which have diets typically low in long chain unsaturated fatty acids. Positive selection has also been demonstrated for genes coding for transporters of zinc, an important cofactor of several enzymes and DNA-binding proteins [11, 12].

The objective of this study was to identify variants showing signs of positive selection in genes coding for cofactor transporters (hereafter referred to as CT and listed in Additional file 1: Table S1). We posit that adaptation to different ecological niches may also select for other genes involved in nutrient transport and metabolism, especially those that affect multiple cellular and biochemical processes such as cofactors or their micronutrient precursors. Cofactor transporter genes may be more susceptible to being influenced by different environments and nutritional habits because of their importance in nutrient absorption and subsequent tissue distribution.

To fulfill this objective, genetic differentiation of CT-associated variants were analyzed using data from the Human Genome Diversity Project (HGDP), a dataset chosen because it includes multiple world populations representative of a variety of environments and ancestral nutritional habits [1, 13, 14]. Using an approached based on principal component analysis (PCA) [15–17], 24 variants in 14 CT genes with signals of positive selection that could contribute to various disease risks and response to nutritional intervention observed between individuals with different genetic makeup were identified.

## Results

### Identification of proteins involved in cofactor transport

Public databases (i.e., NCBI PubMed, UniProt, and OMIM databases) were searched for proteins involved in the transport of cofactors (or their nutrient precursors) between cells or sub-cellular compartments. CTs are a subset of proteins that transport other nutrients such as essential fatty acids or amino acids. At least one transporter was identified for 28 of 43 nutrient-derived cofactors [18] (see the “Methods” section for further details and Additional file 1: Table S1 for full list of cofactors and corresponding transporters). Some of the fat-soluble cofactors such as pyrroloquinoline quinone (PQQ), topaquinone, qbiquinone (CoQ), menaquinone (Vitamin K), and lipoic acid diffuse freely across membranes and are transported in lipoproteins in the blood. Other cofactors, such as biopterin, tetrahydrobiopterin (BH4), molybdopterin (MPT), and *S*-adenosyl-l-homocysteine (SAH), are synthesized in cells and used locally and as such do not require transporters. Fe-S complex, heme-thiolate, inositol hexaphosphate, and dipyrromethane circulate as part of hemoglobin in red blood cells. The gene coding for the pyridoxal phosphate (vitamin B_6_) transporter has not yet been identified [19].

A total of 312 proteins are involved in the transport of cofactors with 39 able to transport more than one cofactor. The transporters with affinity to the most cofactors are the cation transporters CNNM2 (cyclin and CBS domain divalent metal cation transport mediator 2) and NIPAL1 (non-imprinted in Prader-Willi-like domain containing 1) that mediate the trans-membrane movement of five divalent cations—cobalt, copper, iron, magnesium, and manganese.

### Cofactor transporters genetic diversity

**Fig. 1:**
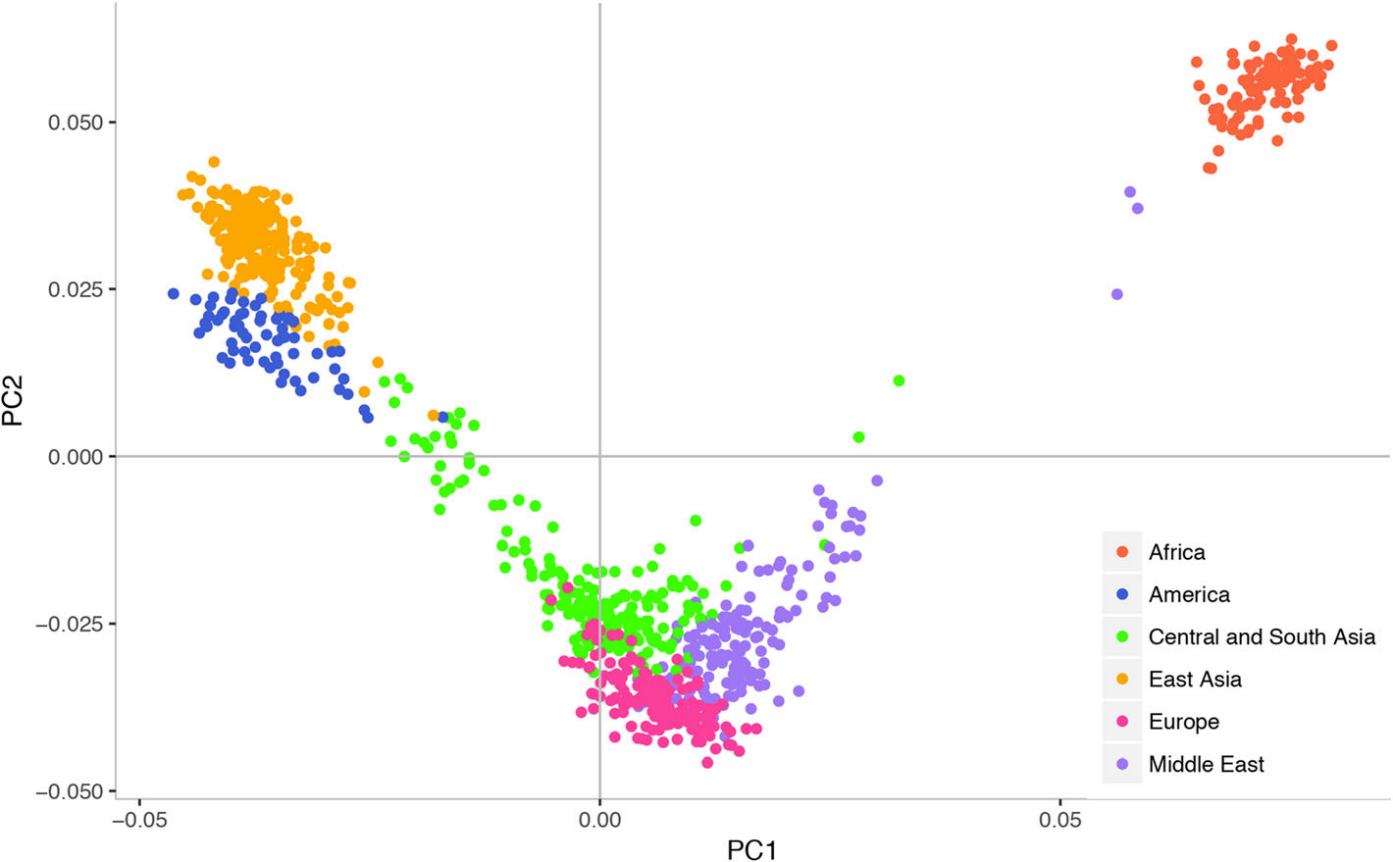
PCA result. The scatterplot shows the first two components of PCA analysis based on genotype data of SNPs located in genes coding for transporters of cofactors in individuals from HGDP dataset. Each point corresponds to one individual, color-coded according to the geographic region of origin as shown in the legend

Genotype data from HGDP was used to study the genetic differentiation in genes coding for CTs. The final sample set included 940 individuals from 53 populations using the quality control criteria described in the “Methods” section. Genetic variation in CT genes was summarized by PCA. During the computation, smartpca removed 27 subjects belonging to Papuan and Melanesian populations because their PC values exceeded 6 standard deviations from population and were deemed as outliers. Nine hundred thirteen individuals were thus included in the following analyses. The percentage of explained variance of each PC is shown in Additional file 2: Figure S1. First three PCs were sufficient to separate the populations into their corresponding continental groups using the genetic variants in CT genes. In particular, PC1 separated African populations from all others, PC2 described a gradient from East Asia to Middle East and Europe, and PC3 divided Native American populations from the others (Fig. 1 and Additional file 3: Figure S2). The subsequent PCs described intra-continental genetic differences. In particular, PC5 and PC6 separated the traditional African hunter-gatherer groups (San, Mbuty Pygmy, and Biaka Pygmy) from the African populations that adopted the agricultural, sedentary lifestyle hereafter referred to as farmers (Bantu from South Africa, Bantu from Kenya, Yoruba, and Mandenka) (Additional file 4: Figure S3). The grouping of subjects observed in the PCA of transporters was similar to the results of PCA performed using genome-wide genotype data (Additional file 5: Figure S4).

### Positively selected SNPs and genes

A methodology based on PCA loadings was used to identify loci under positive selection. This method does not require a priori separation of individuals by population and is thus beneficial with datasets such as the HGDP composed of individuals representing a large spectrum of genetic diversity (see the “Discussion” and “Methods” sections for further details). This method was first tested on the entire genome-wide dataset (Additional file 6: Table S2). The relevance of these findings was evaluated by further looking in the literature for the top 10 loci of each of the first ten PCs. All these loci spanned a region that included a SNP with a *q* value < 0.05, with the exception of the SNPs related to PC1, PC2, and 1 SNP associated to PC6 (rs11682328) that did not exceed this threshold. Sixty-one of these 100 loci corresponded to genes previously described as being positively selected in the dbPSHP database [20] (Additional file 7: Table S3) such as, *OCA2*/*HERC2*, *SLC24A5*, and *EDAR* [21, 22]. The workflow was then applied to the CT dataset. Twenty-four SNPs corresponding to 14 CT genes differentiated along the first five PCs (i.e., PC3 and PC5) (Table 1). The SNPs showing evidences of positive selection in the subsequent PCs are reported in Additional file 8: Table S4. Positive selection in CTs was also evaluated using the integrated Haplotype Score (iHS) selection metrics calculated in HGDP [23] and grouping SNPs at the gene level. Most of the genes previously identified using the PCA workflow, with the exception of *CACNA1A*, *HPX*, *SLC11A2 SLCO1A2*, and *TRPM4*, showed evidence of positive selection in at least one population or group of populations using this method (detailed in the Additional file 9: Note 1).

**Table 1:** Positively selected SNPs within cofactor transporter genes

Genes	Official gene name	Cofactors	Tissue enrichment^a^	Chr	PC	SNPs
CACNA1A	Calcium voltage-gated channel subunit alpha1 A	Ca	Tissue enhanced: cerebral cortex; stomach	19	PC3	rs7254771 (0.03)
CACNB4	Calcium voltage-gated channel auxiliary subunit beta 4	Ca	Tissue enhanced: cerebral cortex	2	PC5	rs16830593 (0.007); rs11902858 (0.02)
HPX	Hemopexin	Fe	Tissue enriched: liver	11	PC5	rs16913549 (0.01)
KCNB2	Potassium voltage-gated channel subfamily B member 2	K	Tissue enhanced: cerebral cortex; spleen	8	PC5	rs7833062 (0.04); rs6996335 (0.02)
KCNH5	Potassium voltage-gated channel subfamily H member 5	K	Tissue enhanced: adrenal gland; cerebral cortex	14	PC5	rs8019319 (0.007)
KCNH7	Potassium voltage-gated channel subfamily H member 7	K	Tissue enriched: cerebral cortex	2	PC3; PC5	rs6753132 (0.05); rs6708255 (0.007); rs7588788 (0.07)
KCNK13	Potassium two pore domain channel subfamily K member 13	K	Tissue enhanced: testis	14	PC3	rs3861656 (0.025); rs4462529 (0.025); rs17223880 (0.025)
LRP2	LDL receptor related protein 2	D3	Group enriched: kidney; placenta; thyroid gland	2	PC5	rs16856593 (0.004)
RYR2	Ryanodine receptor 2	Ca	Tissue enriched: heart muscle	1	PC5	rs12087761 (0.011)
SLC11A2	Solute carrier family 11 member 2	Co	Expressed in all	12	PC5	rs12312876 (2.70E-08)
SLC24A3	Solute carrier family 24 member 3	K,Ca	Mixed	20	PC5	rs10485588 (0.04); rs16980447 (0.03); rs6112335 (0.02); rs6035421 (0.02)
SLC25A26	Solute carrier family 25 member 26	SAM	Expressed in all	3	PC3	rs17044224 (0.03); rs1471476 (0.03);
SLCO1A2	Solute carrier organic anion transporter family member 1A2	GSH	Group enriched: cerebral cortex; liver; lung; salivary gland	12	PC5	rs2199685 (0.03)
TRPM4	Transient receptor potential cation channel subfamily M member 4	Ca	Mixed	19	PC5	rs8104571 (0.0008)

*Ca* calcium, *Co* cobalt, *Chr* chromosome, *D3* vitamin D_3_, *Fe* iron, *K* potassium, *GSH* Glutathione, *PC* principal component, *SAM S*-Adenosylmethionine

^a^Tissue enrichment category from Human Protein Atlas among the following categories: (i) Tissue enriched: mRNA levels in one tissue at least five times higher than all other tissues, (ii) Group enriched: mRNA levels of a group of 2 to 7 tissues at least five times those of all other tissues, (iii) Tissue enhanced: mRNA levels in a particular tissue at least five times the average level in all tissues, (iv) Expressed in all: mRNA detected in all tissues, (v) Mixed: detected in fewer than 32 tissues but not elevated in any tissue, or (vi) Not detected. Tissue(s) where protein is enriched in cases of Tissue enriched, enhanced or group enhanced is listed

### Functional annotation and linkage disequilibrium patterns of positively selected SNPs

SNPs showing signs of positive selection were annotated using Ensembl transcript to investigate their functional consequences within or flanking each gene. None were found in exons (Additional file 10: Table S5). However, four SNPs (rs16830593 in *CACNB4*, rs1471476 and rs17044224 in *SLC25A26*, and rs10485588 in *SLC24A3*) were identified as significant cis-eQTLs from the GTeX eQTL database [24] (Table 2). Moreover, an additional SNP in *SLC24A3* (rs16980447) showed a nominal *p* value < 0.05 but was not significant after FDR correction. *SLC24A3* SNPs were found to be associated with its expression level in blood cells while the *CACNB4* variant was associated with its gene expression level in skin exposed to sun. *SLC25A26* SNPs were cis-eQTL in the heart and adipose tissue. Two SNPs, rs3861656 in *KCNK13* andrs16830593 in *CACNB4*, are likely to affect transcription factor binding (RegulomeDB variant classification of 2b and 2c, respectively) (Additional file 11: Table S6).

**Table 2:** Significant eQTL from positively selected cofactor transporter SNPs

PC	SNP	Gene	Official gene name	Tissue	Cofactors	Effect size	*p* value
3	rs1471476	SLC25A26	Solute Carrier Family 25 (Mitochondrial Carrier; Phosphate Carrier), Member 26	Heart—left ventricle	SAH	− 0.49	1.4E−06
3	rs17044224	SLC25A26	Solute Carrier Family 25 (Mitochondrial Carrier; Phosphate Carrier), Member 26	Adipose—subcutaneous	SAH	− 0.32	4.9E−05
3	rs17044224	SLC25A26	Solute Carrier Family 25 (Mitochondrial Carrier; Phosphate Carrier), Member 26	Heart—left Ventricle	SAH	− 0.5	5.0E−07
5	rs10485588	SLC24A3	Solute carrier family 24 (sodium/potassium/calcium exchanger), member 3	Whole blood	K, Ca	0.74	1.9E−08
5	rs16830593	CACNB4	Calcium Channel Voltage-Dependent Subunit Beta 4	Skin—sun exposed (lower leg)	Ca	− 0.82	6.6E−05

From GTeX eQTL database

*Ca* calcium, *K* potassium, *SAH S*-Adenosyl-l-homocysteine, *PC* principal component

**Fig. 2:**
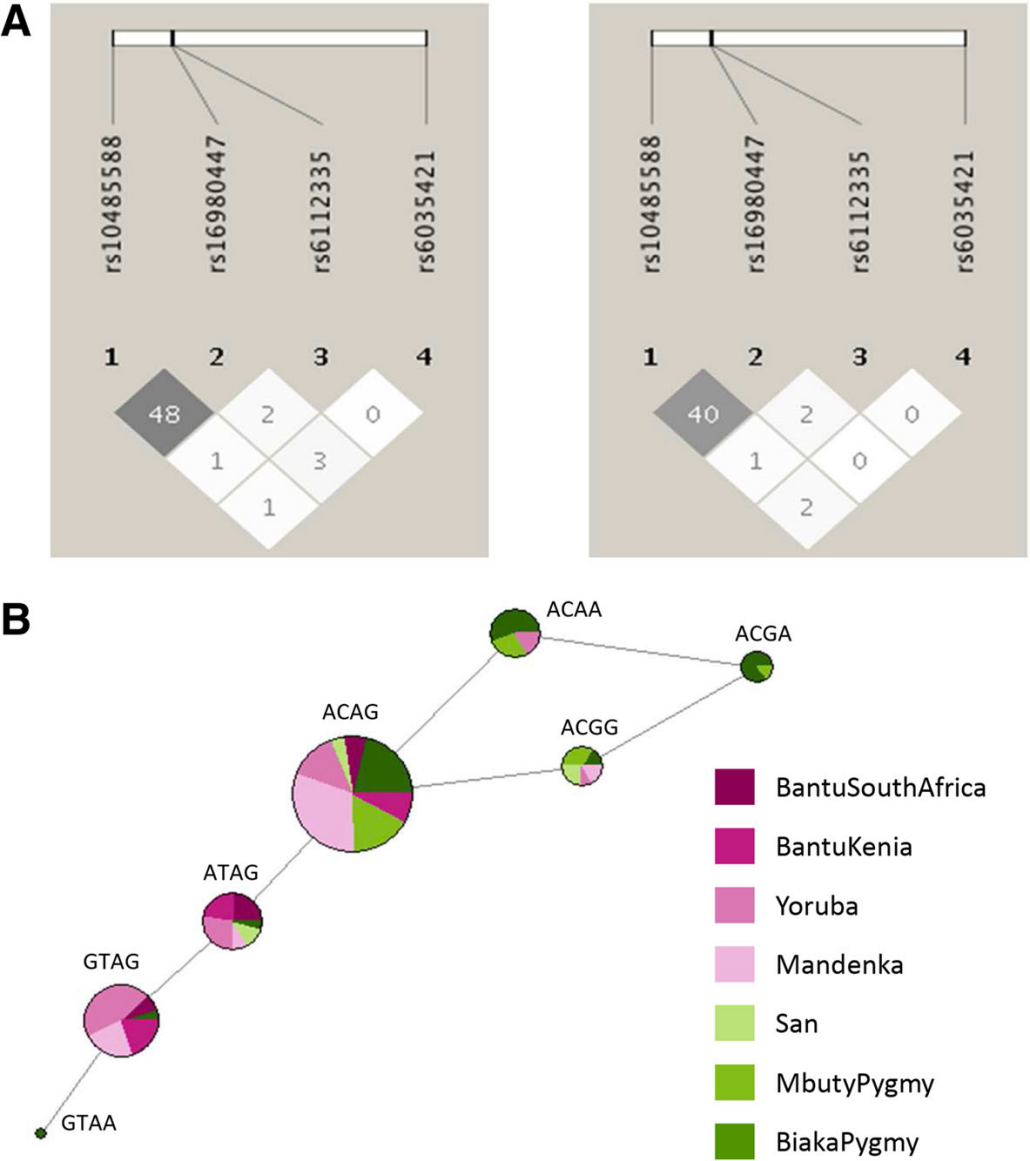
Linkage disequilibrium plots and haplotype network of *SLC24A3* regions in African populations. **a** Visualization of LD between the genetic variants in *SLC24A3* regions bearing signals of positive selection. LD was calculated using *r* ^2^ parameter separately in African populations of farmers and hunter-gatherers. Squares shaded according to strength of LD. **b** Haplotype network analysis of *SLC24A3* regions. Each circle represents a haplotype that is color-coded according to the population in which it is present. Circle sizes are proportional to the haplotype frequency and each line corresponds to one mutational step

**Fig. 3:**
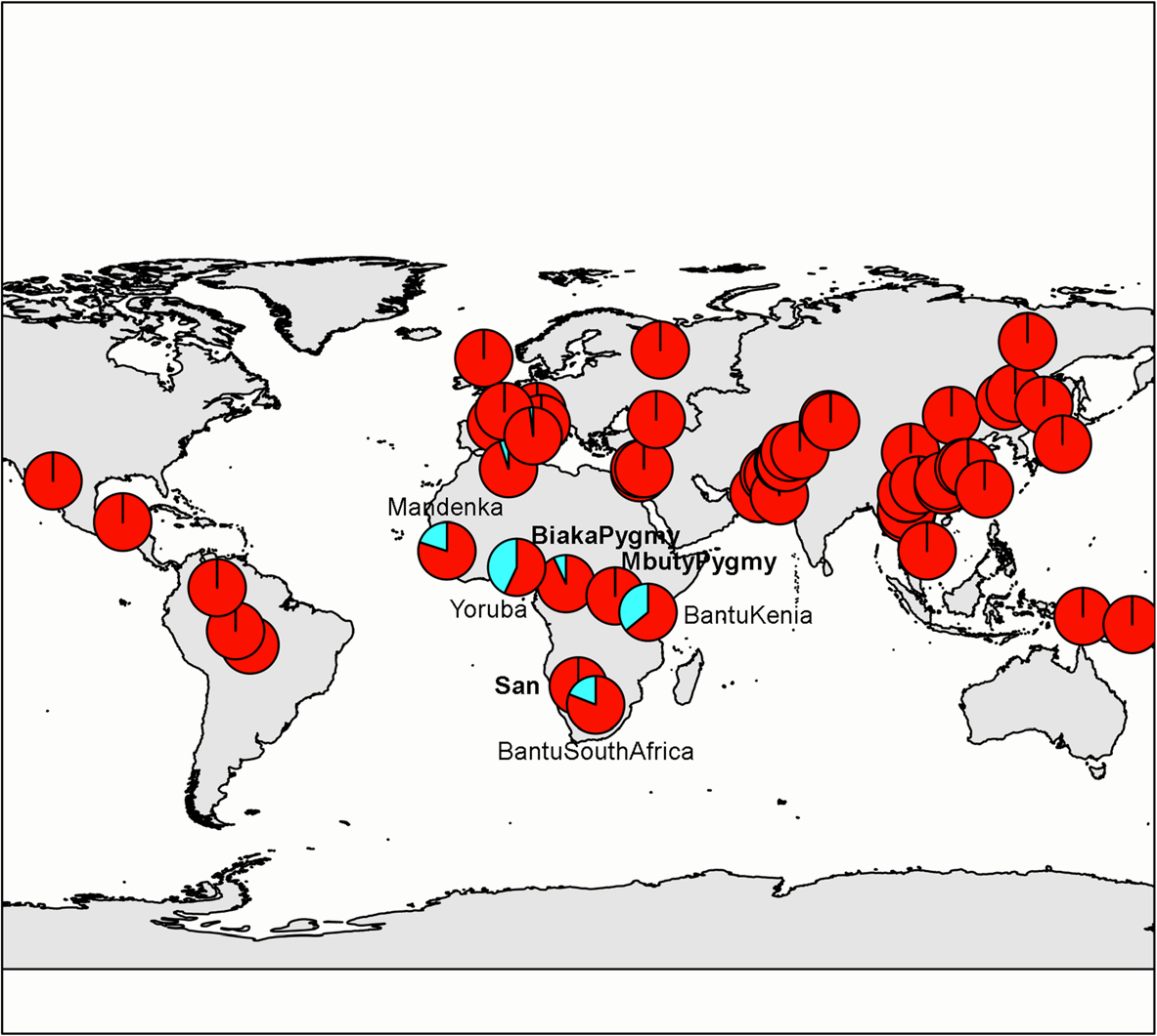
Spatial frequency distribution of rs10485588 alleles. Each pie chart corresponds to one HGDP population and is positioned on the map according to the latitude and longitude data used by Rosenberg et al. [44]. Pie charts are colored according to the frequency of the common, ancestral A (red) and the derived G (light blue) alleles. Note that among the African populations, hunter-gatherers are written in bold

Proxy SNPs using the Yoruba population from the 1000 Genomes database were used to investigate whether non-mapped functional SNPs were in linkage disequilibrium (LD) with SNPs differentiated in African populations (related to PC5). No non-synonymous SNPs were found among those in LD with the differentiated SNPs (*R*-square > 0.8). However, two missense SNPs were identified as proxy SNPs (rs6757850 correlated with *KCNH7* SNP rs6708255 and rs7588788 and rs114005357 correlated with *SLC11A2* SNP rs12312876) when lowering the *R*-square threshold to 0.4. Similar analysis was not possible for Native American populations since no sequencing data from a different dataset was available to evaluate LD. For what concern PC5, we observed that the clustering of African populations in two groups corresponded to one of the two subsistence strategies traditionally adopted by these populations, namely being primarily farmers or hunter-gatherers. The best candidate gene related to PC5 is *SLC24A3* since it contains four SNPs showing evidences of positive selection, one of which also being a strong eQTL in GTeX database. The African genetic variation in the *SLC24A3* region was further examined by estimating haplotypes to better evaluate the difference in allele frequencies of *SLC24A3* region between the previously identified groups of farmers and hunter-gatherers. The most common haplotype is characterized by the SNP alleles ACAG shared by both farmers and hunter-gatherers. Notably, some haplotypes were restricted to only one sub-group (Fig. 2b). Specifically, the haplotype GTAG was separated from the network core by rs10485588 (A [red in Fig. 3] and G [blue in Fig. 3], the ancestral and derived alleles, respectively), the putative eQTL SNP, which is found predominantly in farmer populations (with the exception of two Biaka Pygmies individuals) (Fig. 3). The haplotype with the alternative alleles for those SNPs (i.e., ACGA) is completely absent among farmers.

## Discussion

Positive selection of genes coding for proteins involved in cofactor transport between cells or sub-cellular compartments was found by comparing genotypes of populations from the HGDP. This dataset is particularly interesting since it includes genotypes from several genetically diverse worldwide populations, whom ancestries have evolved in different environments and thus been exposed to diets of varying nutritional composition (i.e., hunter-gatherers and farmers). Cofactor transporters are of particular interest as they regulate the tissue and sub-cellular bioavailability of micronutrient-derived cofactors and are more likely to be influenced by different nutritional habits from ancient populations originating from regions with varying climates [1] and soil composition [25]. Cofactor-requiring biological processes participate in normal and pathophysiological processes that could contribute to between-population differences in disease incidence and response to nutritional interventions and diets [18, 26]. However, other selective forces may have contributed to the evolution and distribution of CT variants among populations.

The PCA-based approach followed here associated the population-specific alleles to a specific PC and thus a specific ancestry gradient. Contrarily to F_ST_ statistic, a popular measure of positive selection based on population differentiation [27], it does not require a priori definition of populations or groups of populations [16]. We thus considered it more suitable for the HGDP dataset, which contains several populations and some of them not being genetically well separated from one another. Moreover, since the PCA-based approach identifies outlier SNPs for each principal component, it is less likely to identify variants that underwent random genetic drift since such phenomenon should similarly affect all variants in a population.

The signals of positive selection identified here were derived mainly from two PCs, namely PC3 and PC5. The gradient described is intra-continental and is due to the difference in allele frequencies across the Native Americans and Africans populations, respectively. PC5 separated African hunter-gatherers from farmers, two populations that traditionally based their subsistence on different diets and identified *SLC24A3* as being positively selected. *SLC24A3* encodes for the potassium-dependent Na+/Ca2+ exchanger type 3 protein (NCKX3), an important regulator of intracellular calcium homeostasis. This gene is expressed most abundantly in the brain but also found in the aorta, uterus, intestine, and skeletal muscle with low expression in other tissues [28].

Polymorphisms in *SLC24A3* have been associated with salt-sensitive vasoconstriction and hypertension [29], while the expression of NCKX3 protein was linked to preeclampsia (i.e., pregnancy complicated by high blood pressure) [30]). Selection of these variants in hunter-gatherers may be due to diverse, animal-based, diets that were low in sodium chloride and high in potassium salt intake compared with the diet adopted after the Neolithic transition [31]. Indeed, this transition took place at the end of the most recent ice age and coincided with the advent of agriculture which was characterized by increases in plant-based at the expense of animal-based ingredients and where salt became an important commodity. Adaptation to such dietary pattern must have induced genetic adaptation in many genes involved in nutrient metabolism and may partly explain modern-day phenotypes, as that observed recently with the FADS gene [10, 32]. Namely, individuals with varying admixture from hunter-gatherers to farmers, such as modern Europeans [33], have different risks of cardiovascular disease, hypertension, stroke, kidney stones, and osteoporosis (e.g., [34]) compared to African-Americans (e.g., [35]), which could be mediated by their different metabolic response to various dietary minerals. In fact, a short-term intervention with a hunter-gatherer, or Paleolithic, diet improved glucose homeostasis and lipid profiles in modern-day Americans living with type II diabetes [36]. The opposite is also possible to envision. Namely, transitioning from a hunter-gatherer to a post-Neolithic diet could induce metabolic alterations that, in longer-terms, would increase cardiovascular and other chronic disease risks.

Limitations were inevitably present in the study and should be considered when interpreting observations. First, the HGDP dataset, obtained using the DNA chip technology, does not allow studying rare variants that would instead be detected using newer technology such as the next generation sequencing. Moreover, each population in the dataset is represented by a small sample and could be the reason of not having extremely significant results. In fact, even if all the SNPs reported in the manuscript were significant after FDR correction, only one met the genome-wide significance threshold of *p* < 5 × 10^−8^, rs12312876 (*p* = 2.70 × 10^−8^). This issue could be overcome using 1000 Genomes dataset; however, the populations included in that project do not cover the spectrum of human genetic differentiation that would be necessary to study the selective pressure exerted by diet. In fact, even close populations such as the African farmers and hunter-gatherers, not present in 1000 Genomes, could have been affected by different environmental factors. Another important limitation is the lack of direct information on dietary habits of reference populations that prevent any conclusion about the driving force of the adaptation. For what concern the analysis, the method we chose allowed us not to split the dataset in separate populations and thus has been the methodology of choice. However, if we had the possibility of using larger sample sizes for the population of interest, it would have been interesting to apply other selection metrics such as the haplotype-based methods such as iHS [37] and XP-EHH [38], calculated for each population instead of groups of populations, or the XP-CLR method [39], which uses allele frequency differentiation between populations to detect selective sweeps. The availability of sequencing data would allow to test also other methods, such as the population branch statistic (PBS), which was successful in identifying genes involved in adaptation to high altitude from exome sequencing data [40].

## Conclusion

Genetic variation in cofactor transporters may be of use clinically to investigate and help explain inter-individual variability in response to dietary interventions [18]. Indeed, individual CT SNP distribution, reflective of their genetic backgrounds, could influence the expression or activity of these important mediators of micronutrient-derived cofactor ADME and biological effect. Thus, our findings support the importance of considering an individual’s genetic makeup along with their metabolic profiles (e.g., homeostatic measures of vitamin levels for instance) when tailoring and analyzing responses to personalized dietary interventions aimed at optimizing health.

## Methods

### Cofactor and transporter identification

NCBI PubMed (http://www.ncbi.nlm.nih.gov/pubmed), UniProt (http://www.uniprot.org/), and OMIM (http://www.omim.org/) databases were searched for transporters of cofactors [18]. The cofactor name and their synonyms with the addition of the word “transport” or “transporter” were used for the PubMed search. For instance, combinations of one of the following vitamin C synonyms “vitamin C”, “vit C”, “ascorbic acid”, and “ascorbate” AND “transporter” were searched to identify vitamin C transporters. The transporters identified from NCBI PubMed were verified on the UniProt database for their involvement in the transport of other cofactors.

Tissue-specific expression of CTs was evaluated using data extracted from the Human Protein Atlas database, which classifies proteins into the following categories: (i) Tissue enriched: mRNA levels in one tissue at least five times higher than all other tissues, (ii) group enriched: mRNA levels of a group of 2 to 7 tissues at least five times those of all other tissues, (iii) tissue enhanced: mRNA levels in a particular tissue at least five times the average level in all tissues, (iv) expressed in all: mRNA detected in all tissues, (v) mixed: detected in fewer than 32 tissues but not elevated in any tissue, or (vi) not detected (resulting tissue-specific information can be found in Additional file 1: Table S1) [41].

### Genetic variation data

The genotype data were obtained from the HGDP–CEPH panel, a resource that captures a significant proportion of human genetic diversity. The genotypes were obtained with the Illumina BeadStation technology for 1043 individuals, were downloaded from http://www.hagsc.org/hgdp/files.html, and were pre-processed at the SNP and individual levels using PLINK v1.07 [42]. Before the quality control procedure, 660,918 SNPs were available. Sixteen thousand six hundred fifty non-autosomal SNPs and 1248 SNPs with a genotyping rate less than 0.95 and 12,085 SNPs with a minor allele frequency less than 0.01 were excluded for a total of 630,935 remaining SNPs (of which 8960 SNPs for CT genes). Additionally, 103 related individuals from both first- and second-degree relative pairs, as described in Rosenberg, 2006 [43], were also discarded. The assignment of individuals to populations was performed using the table downloaded from the Rosenberg Lab website http://rosenberglab.stanford.edu/data/rosenberg2006ahg/SampleInformation.txt, as published in Rosenberg, 2006 [43]. According to this data the HGDP individuals were assigned to 53 populations. The geographic coordinates were downloaded from the same web source (https://web.stanford.edu/group/rosenberglab/data/rosenbergEtAl2005/rosenbergEtAl2005.coordinates.txt), and they have been previously used in Rosenberg et al. [44].

### Principal component analysis

Principal component analysis was performed with smartpca tool of the EIGENSOFT package v6.0.1 [45] using the default settings that allow the removal of individuals detected as outliers during the computation. A preliminary PCA on the genome-wide data was used as an additional quality control step to detect the presence of outliers or individuals not grouped with their geographic region of origin, and we did not detect any issue. Next, we used PCA to evaluate the population stratification both at genome-wide level and on CT genes only. The pattern of differentiation in CT genes was investigated on a subset of 8960 SNPs located in CT genes [46, 47].

### Selection statistic

Statistical analyses were performed with R 3.1.2 (R Foundation for Statistical Computing, Vienna, Austria; http://www.r-project.org/) unless otherwise specified. Our analysis was designed to identify SNPs with signal of positive selection on the basis of outlier detection from principal component analysis. Such PCA-based approaches were recently successful in identifying genetic loci under adaptive selection [15–17]. The main advantage of this approach over other methods like F_ST_ statistic is that it assesses genetic differentiation along gradients without requiring a priori clustering of the individuals by population. Starting from the SNP weights (loadings) obtained from the smartpca output, the selection statistics *D* ^2^ was calculated and it corresponds to the squared loading of each SNP [15, 16]. The discrepancy between the empirical distribution and the theoretical one was determined, and the pchisq R function was used to associate a *p* value to each SNP. *p* values obtained were corrected for multiple testing using the R package *q* value, which controls for false discovery rate (FDR) [48]). *q* value significance threshold of 0.05 was used. To evaluate the results obtained applying the selection statistic to the genome-wide HGDP dataset, the top ten SNPs were extracted for the first ten PCs (100 total SNPs). A genomic region spanning 200 kb around each SNP was identified and genes annotated using the Bioconductor annotation package TxDb.Hsapiens.UCSC.hg18.knownGene. The comparison of results with literature was done using the data from dbPSHP, a database which contains information about genes and genomic regions from curated publications about positive selection in different human populations [20].

### Linkage disequilibrium and haplotype analysis

The identification of the proxy SNPs of each significant variant associated to PC5 was performed using the genotype data of 1000 genomes Yoruba population. The analysis was carried out using the online tool LDlink (https://analysistools.nci.nih.gov/LDlink/?tab=home). We submitted the significant SNPs identified, and for each of them, we retrieved a list of proxy variants located −/+ 500 Kb of the query variant with a pairwise *R* ^2^ value greater than 0.01.

The pattern of LD in *SLC24A3* gene was estimated using Haploview v4.2. The haplotype phase was inferred using fastPHASE v1.4.8. The input files were created using PLINK, and the tool was run using these parameters: 25 iterations of the EM algorithm (C parameter) and 200 as the number of the number of haplotypes sampled from the “posterior” distribution obtained from a particular random start of the EM algorithm (H parameter). To build the haplotype network, we used the indiv.out file which contains estimates which attempt to minimize individual error. The haplotype network was produced by Network 4.2.0.1 using the median-joining algorithm [49].

### Functional annotation

The impact of SNPs on protein function was examined using the Ensembl Variant Effect Predictor tool (http://www.ensembl.org/Homo_sapiens/Tools/VEP/), using the GRCh38.p7 human assembly. The regulatory potential of the SNPs was investigated using the RegulomeDB, Version 1.1 [50]. The data from GTEx database V6 [24] (http://www.gtexportal.org/home/) were used to investigate the presence of correlations between the SNPs and tissue-specific gene expression levels (i.e., eQTL).

## Additional files

Additional file 1: Table S1. List of all proteins identified as transporters of cofactors. (XLSX 174 kb)

Additional file 2: Figure S1. Scree plot from PCA. This chart shows the eigenvalues associated with each PC. (PDF 6 kb)

Additional file 3: Figure S2. PCA analysis for PC3 and PC4. The two scatter plots show the grouping of individuals according to PC1/PC3 and PC1/PC4. (PDF 17 kb)

Additional file 4: Figure S3. PCA analysis for PC5, 6, and 8. Principal components showing positive selection between African sub-populations of Hunter-gatherers and Farmers (PC5/6) and between Native Americans (PC5/8). (PDF 19 kb)

Additional file 5: Figure S4. PCA analysis of entire HGDP dataset. The scatter plots show the grouping of individuals according to PC1 and PC2 using all the autosomal SNPs. (PDF 10 kb)

Additional file 6: Table S2. Analysis of positive selection using the GWAS dataset and literature comparison. (XLSX 298 kb)

Additional file 7: Table S3. SNP functional annotation. (XLSX 194 kb)

Additional file 8: Table S4. Regulome DB annotation. (XLSX 59 kb)

Additional file 9: Note 1. (DOCX 32 kb)

Additional file 10: Table S5. SNP functional annotation (XLSX 13 kb)

Additional file 11: Table S6. Regulome DB annotation. (XLSX 10 kb)

## Abbreviations

CT: Cofactor transporter
HGDP: Human genome diversity project
IHS: Integrated haplotype score
PC: Principal components
PCA: Principal component analysis
