# Supplementary material for: Ancestors’ dietary patterns and environments could drive positive selection in genes involved in micronutrient metabolism—the case of cofactor transporters
Source: Genes Nutr. 2017 Oct 4;12:28. doi: 10.1186/s12263-017-0579-x (PMC5628472; doi:10.1186/s12263-017-0579-x)
Supplement: Supplementary file 2 — Scree plot from PCA. This chart shows the eigenvalues associated with each PC. (PDF 6 kb) [file 12263_2017_579_MOESM2_ESM.pdf]

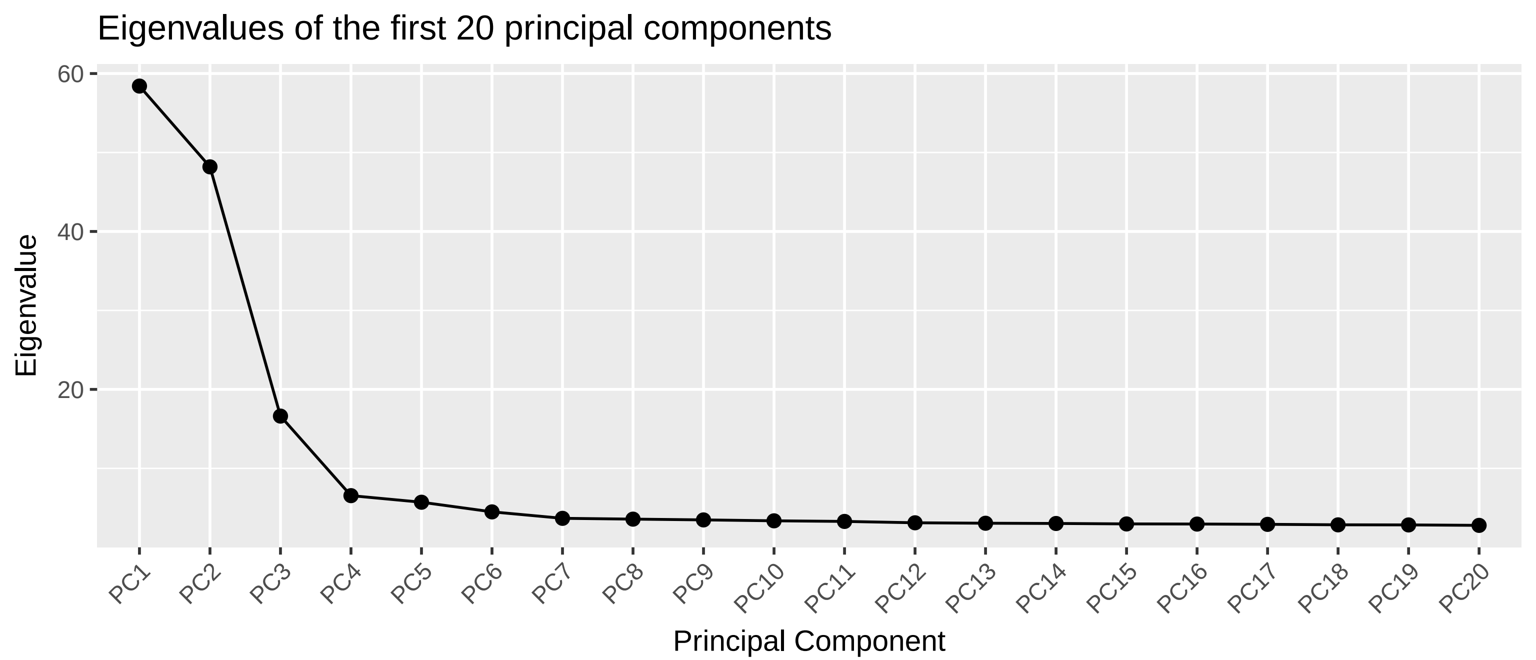
Eigenvalues of the first 20 principal components
Eigenvalue
60
40
20
PC1
PC2
PC3
PC4
PC5
PC6
PC7
PC8
PC9
PC10
PC11
PC12
PC13
PC14
PC15
PC16
PC17
PC18
PC19
PC20
Principal Component

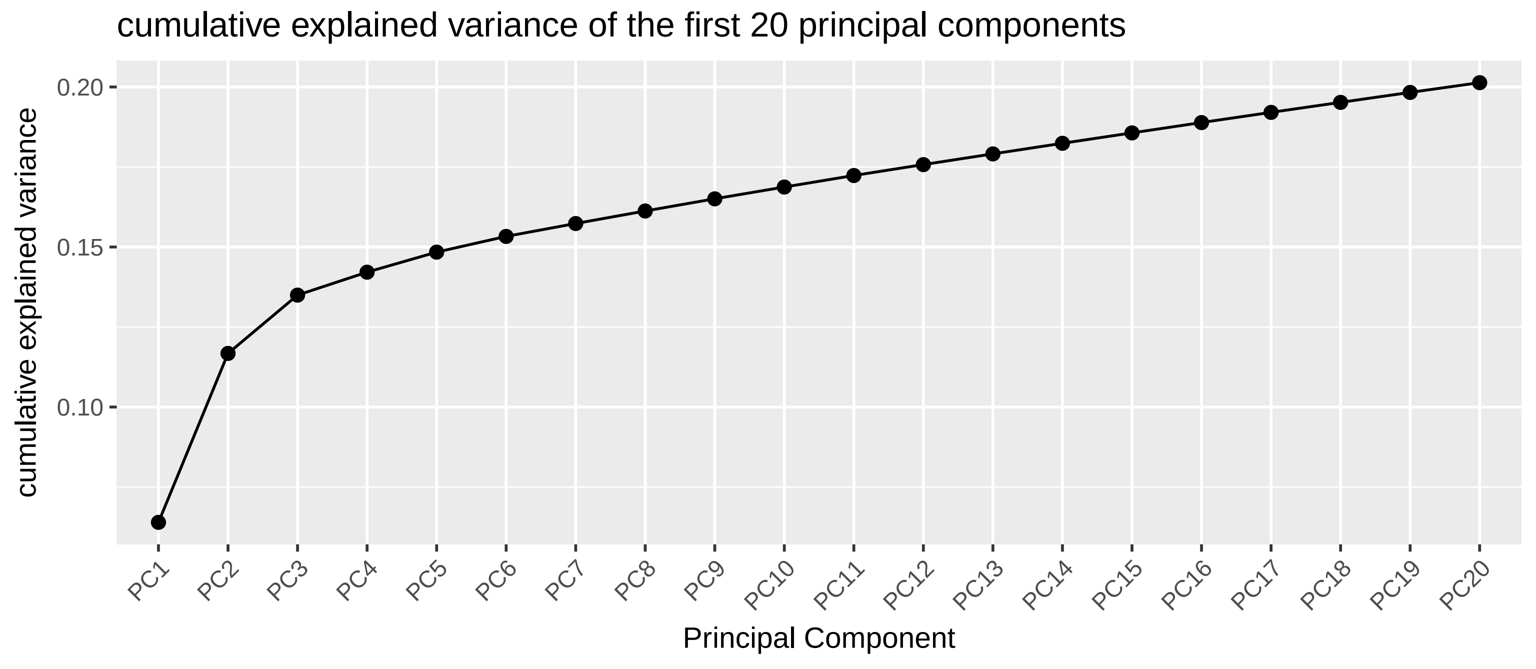
cumulative explained variance of the first 20 principal components
cumulative explained variance
0.20
0.15
0.10
PC1
PC2
PC3
PC4
PC5
PC6
PC7
PC8
PC9
PC10
PC11
PC12
PC13
PC14
PC15
PC16
PC17
PC18
PC19
PC20
Principal Component
