# Supplementary figures and images for: Ancestors’ dietary patterns and environments could drive positive selection in genes involved in micronutrient metabolism—the case of cofactor transporters
Source: Genes Nutr. 2017 Oct 4;12:28. doi: 10.1186/s12263-017-0579-x (PMC5628472; doi:10.1186/s12263-017-0579-x)

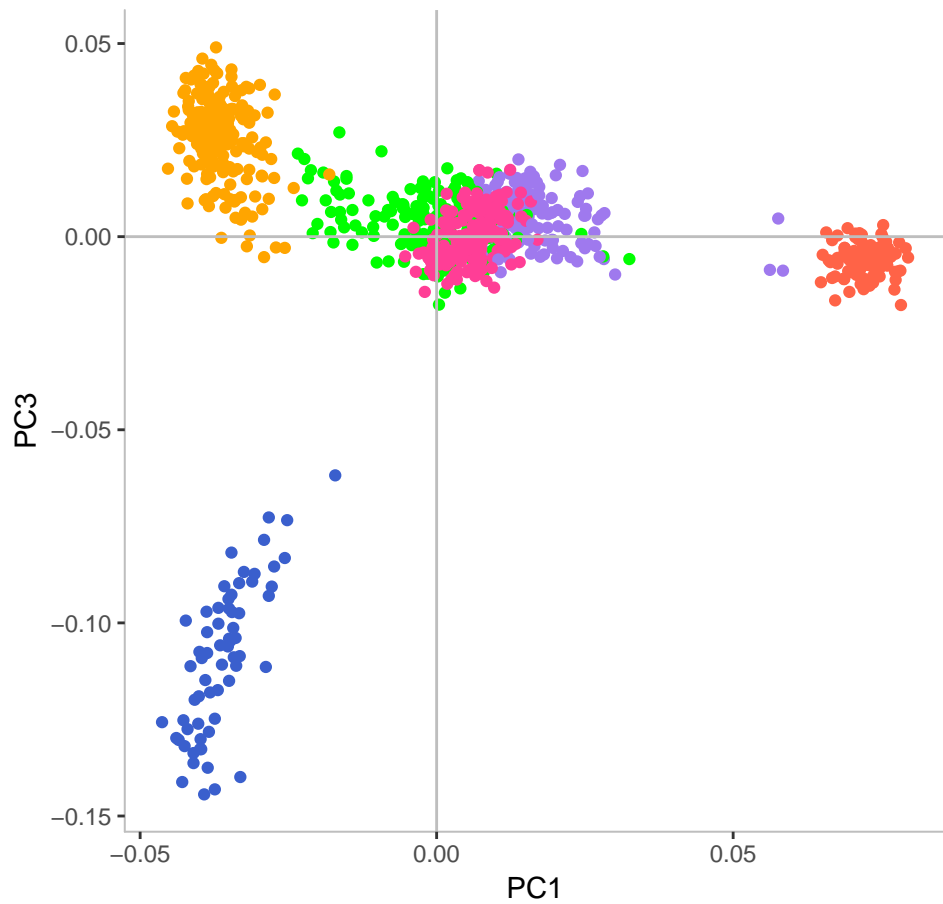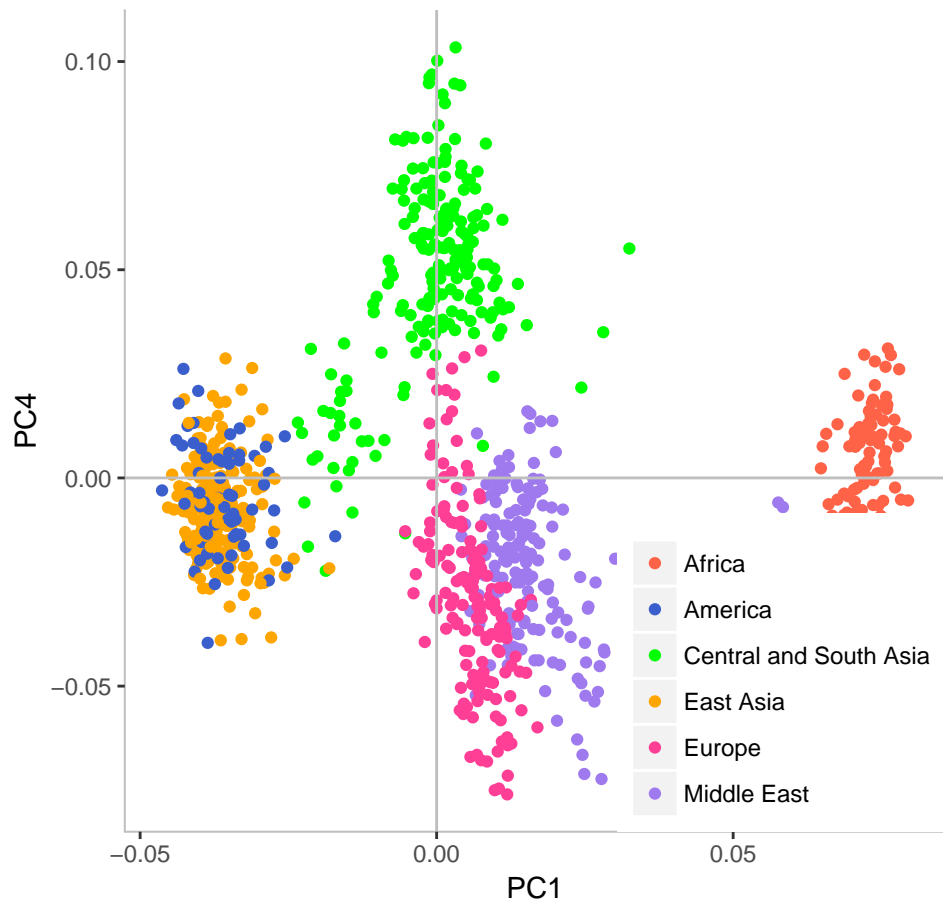

Supplement: Supplementary file 3 — PCA analysis for PC3 and PC4. The two scatter plots show the grouping of individuals according to PC1/PC3 and PC1/PC4. (PDF 17 kb) [file 12263_2017_579_MOESM3_ESM.pdf]

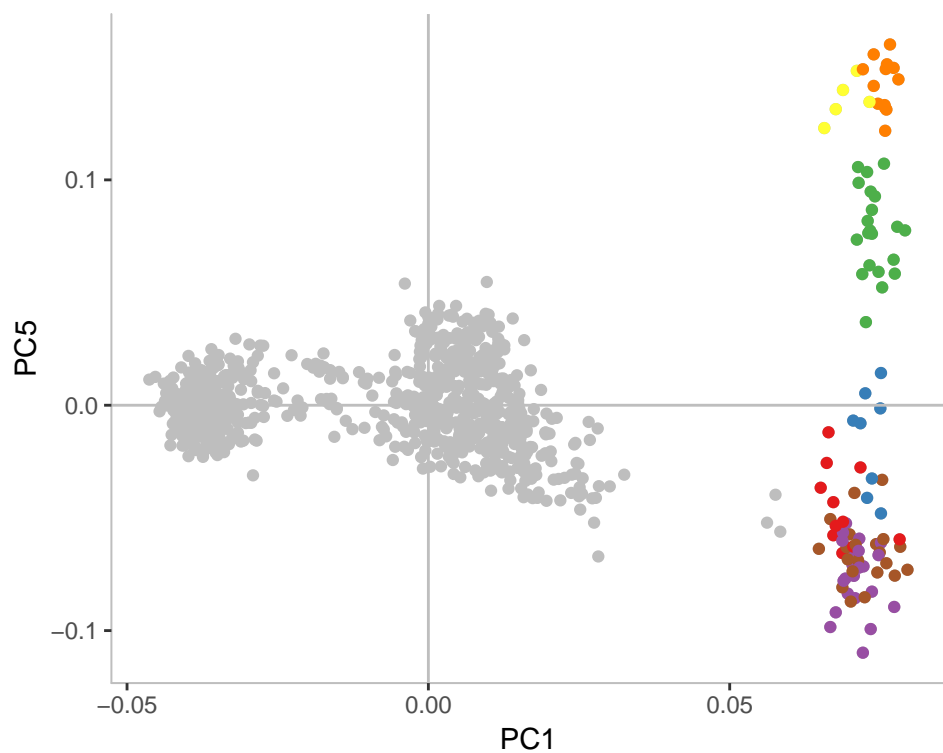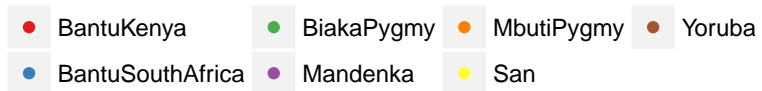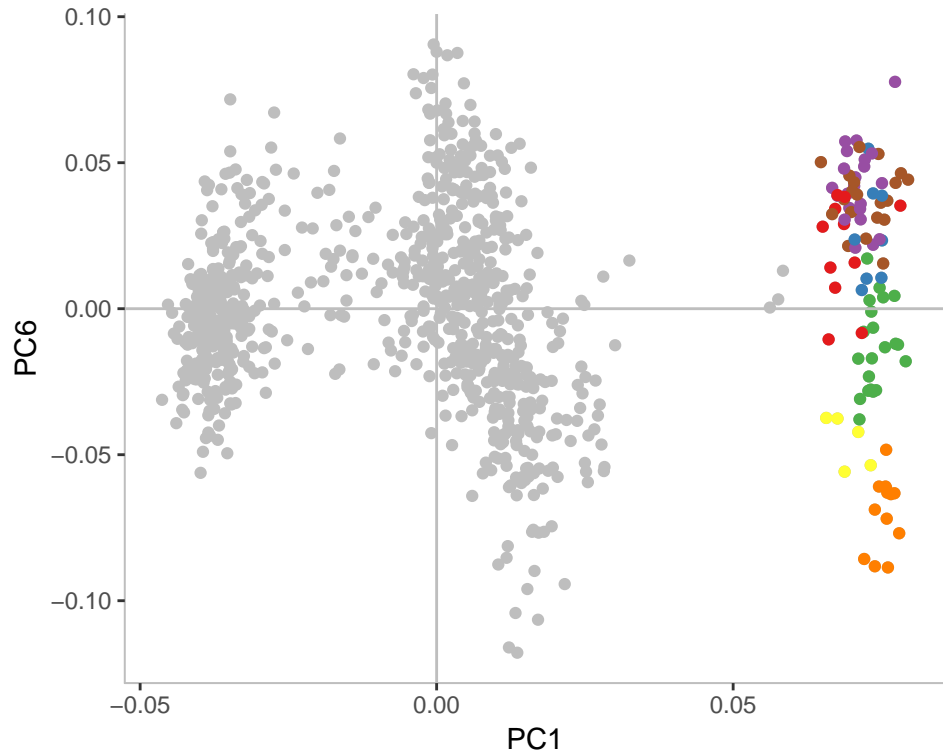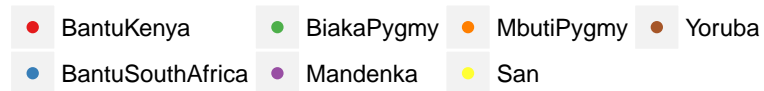

Supplement: Supplementary file 4 — PCA analysis for PC5, 6, and 8. Principal components showing positive selection between African sub-populations of Hunter-gatherers and Farmers (PC5/6) and between Native Americans (PC5/8). (PDF 19 kb) [file 12263_2017_579_MOESM4_ESM.pdf]

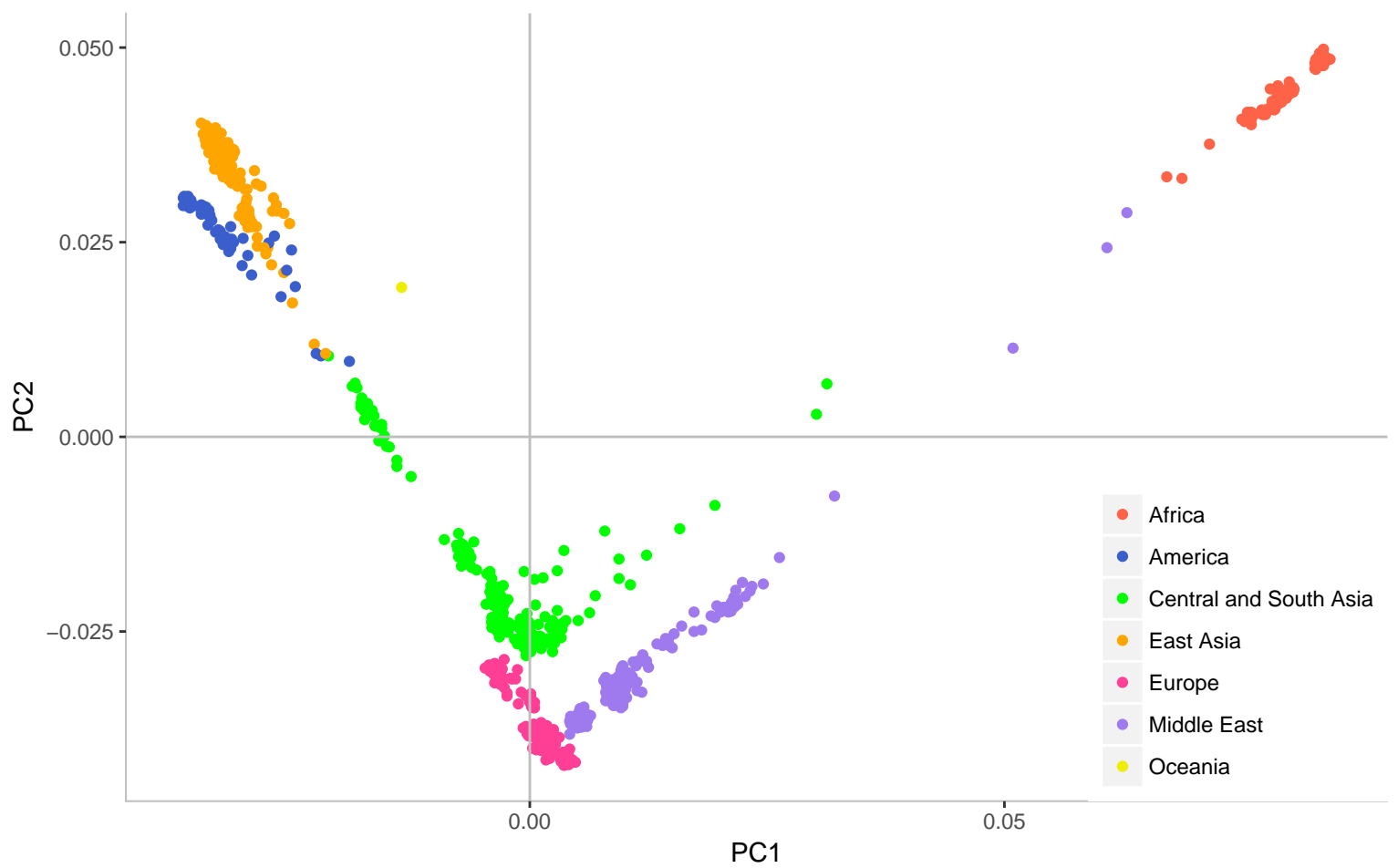

Supplement: Supplementary file 5 — PCA analysis of entire HGDP dataset. The scatter plots show the grouping of individuals according to PC1 and PC2 using all the autosomal SNPs. (PDF 10 kb) [file 12263_2017_579_MOESM5_ESM.pdf]
