# Supplementary material for: Ancestors’ dietary patterns and environments could drive positive selection in genes involved in micronutrient metabolism—the case of cofactor transporters
Source: Genes Nutr. 2017 Oct 4;12:28. doi: 10.1186/s12263-017-0579-x (PMC5628472; doi:10.1186/s12263-017-0579-x)
Supplement: Supplementary file 9 — Note 1. (DOCX 32 kb) [file 12263_2017_579_MOESM9_ESM.docx]

## Integrated Haplotype Score analysis

A complementary approach to PCA, integrated Haplotype Score (iHS) selection metric, was used to test for all genes showing evidence of positive selection in HGDP population [1]. The HGDP populations in this dataset are grouped: Bantu, Biaka, Native Americans, East Asia, Europe, Middle East, South Asia and Oceania.

This selection metric was first described in Voight et al., 2006 [2] and was used in Pickrell et al., to analyze the HGDP dataset [1] because of its good power to detect selective sweeps at moderate frequency. iHS calculation is based on the analysis of haplotypes and it is a measure of the Extended Haplotype Homozygosity (EHH) decay around a SNP comparing the ancestral and the derived allele.

An iHS value was calculated for each gene taking the maximum value in each gene region. The gene regions were defined using the Bioconductor annotation table TxDb.Hsapiens.UCSC.hg18.knownGene. To evaluate if the iHS of transporter genes was significantly higher, a random dataset was used with the same number of genes present in the list of transporters. The iHS of transporters was considered significant when was greater than the 0.95 quantile of the random genes iHS distribution.

In the table below are reported the results obtained for the 14 genes with SNPs showing allele differentiation in PCA analysis.

| PC | | GENE | America | Bantu | Biaka pygmy | East asia | Middle east | oceania | South asia | europe |
| --- | --- | --- | --- | --- | --- | --- | --- | --- | --- | --- |
| PC3/PC5 | KCNH7 | | 1 |  |  |  | 1 |  | 1 |  |
| PC3 | SLC25A26 | | 1 | 1 |  | 1 | 1 |  | 1 | 1 |
| PC3 | KCNK13 | |  |  |  | 1 |  |  |  |  |
| PC3 | CACNA1A | |  |  |  |  |  |  |  |  |
| PC5 | RYR2 | | 1 |  | 1 |  |  | 1 |  |  |
| PC5 | CACNB4 | |  |  |  | 1 | 1 |  |  | 1 |
| PC5 | LRP2 | |  | 1 | 1 |  |  |  |  |  |
| PC5 | KCNB2 | |  | 1 | 1 | 1 |  |  |  |  |
| PC5 | HPX | |  |  |  |  |  |  |  |  |
| PC5 | SLC11A2 | |  |  |  |  |  |  |  |  |
| PC5 | KCNH5 | |  | 1 |  |  | 1 |  | 1 | 1 |
| PC5 | TRPM4 | |  |  |  |  |  |  |  |  |
| PC5 | SLC24A3 | |  | 1 |  | 1 |  |  |  |  |
| PC5 | SLCO1A2 | |  |  |  |  |  |  |  |  |

1. Pickrell JK, Coop G, Novembre J, Kudaravalli S, Li JZ, Absher D, et al. Signals of recent positive selection in a worldwide sample of human populations. Genome Res. 2009;19:826–37.

2. Voight BF, Kudaravalli S, Wen X, Pritchard JK. A Map of Recent Positive Selection in the Human Genome. PLoS Biol. 2006;4:e72.
